# High-dose chemotherapy with autologous stem cell rescue in children under 5 years of age with central nervous system embryonal tumors: results from a prospective cohort in an upper-middle-income country

**DOI:** 10.1007/s00381-026-07367-w

**Published:** 2026-06-30

**Authors:** Andrea Maria Cappellano, Natália Dassi, Francine Tesser Gamba, Victor Gottardello Zecchin, Renata Fittipaldi da Costa Guimarães, Adriana Seber, Rui Manuel Reis, Ana Carolina dos Santos Torquato, Jessica Benigno Rodrigues, Murilo Bonatelli, Patricia Alessandra Dastoli, Marcos Devanir Silva da Costa, Sergio Cavalheiro, Ronaldo Modesto de Souza Filho, Frederico Adolfo Silva, Silvia Regina Caminada de Toledo, Nasjla Saba Da Silva

**Affiliations:** 1 Department of Pediatric Oncology, IOP-GRAACC-Federal University of Sao Paulo, Pedro de Toledo Street, 572, Sao Paulo, SP 04039-001 Brazil; 2 Genetics Laboratory, IOP-GRAACC-Federal University of Sao Paulo, Sao Paulo, Brazil; 3 National Science and Technology, Institute for Children’s Cancer Biology and Pediatric Oncology (INCT BioOncoPed), Sao Paulo, Brazil; 4 Hospital Beneficência Portuguesa de Sao Paulo, Sao Paulo, Brazil; 5 Hospital das Clínicas da Faculdade de Medicina de Ribeirao Preto, Sao Paulo, Brazil; 6 Department of Bone Marrow Transplantation, IOP-GRAACC-Federal University of Sao Paulo, Sao Paulo, Brazil; 7 Hospital Do Cancer de Barretos, Sao Paulo, Brazil; 8 Department of Neurosurgery Oncology, IOP-GRAACC-Federal University of Sao Paulo, Sao Paulo, Brazil; 9 Department of Pathology Oncology, IOP-GRAACC-Federal University of Sao Paulo, Sao Paulo, Brazil; 10 Department of Radiology Oncology, IOP-GRAACC-Federal University of Sao Paulo, Sao Paulo, Brazil

**Keywords:** Autologous stem cell rescue, Brain tumors, Medulloblastoma, Embryonal brain tumors, Molecular alterations

## Abstract

**Purpose:**

High-dose chemotherapy (HDCT) with autologous stem cell rescue (ASCR) has been employed to mitigate the long-term side effects of radiotherapy and improve survival outcomes in infants with embryonal central nervous system (CNS) tumors.

**Methods:**

This prospective study, supported by federal funding from the National Oncological Care Support Program, included children younger than 5 years of age with newly diagnosed CNS embryonal tumors treated with surgery and HDCT/ASCR.

**Results:**

Between 2016 and 2019, 36 patients were included: 22 with medulloblastoma (MB) and 14 with other CNS embryonal tumors. In the MB cohort, the mean age was 2.5 years, 13 were male. Gross total resection (GTR) was achieved in 18 patients; 15 had non-metastatic disease (M0). Among non-MB patients, the mean age was 2.7 years, 11 were female. Nine patients had GTR; 12 had M0. According to the MB molecular subgroup, the 2- and 5-year event-free survival (EFS) rates for the SHH subgroup were 76.9%, and overall survival (OS) was 92.3%. For the non-WNT/non-SHH subgroup, the 2- and 5-year EFS rates were 33.3% and 22.2%, and OS, 66.7% and 55.6%, respectively. Two SHH and five non-WNT/non-SHH patients were successfully salvaged with craniospinal irradiation after a mean of 12 months (range, 7–36 months). Among non-MB patients, the 2- and 5-year EFS and OS were 28.6/19.0% and 42.9/32.1%, respectively. All patients developed grade 3–4 mucositis/typhlitis and febrile neutropenia and 13.9% invasive fungal infections during HDCT.

**Conclusion:**

HDCT/ASCR is feasible in a middle-income country setting and provides favorable outcomes in SHH medulloblastoma. However, prognosis remains poor for non-SHH MB and other embryonal tumors, highlighting the need for novel molecularly guided therapies.

**Supplementary Information:**

The online version contains supplementary material available at 10.1007/s00381-026-07367-w.

## Introduction

Medulloblastoma (MB), atypical teratoid/rhabdoid tumor (AT/RT) and embryonal tumor with multilayered rosettes (ETMR), C19MC-altered, are highly aggressive embryonal tumors that predominantly affect young children [1]. According to the World Health Organization (WHO) 2021 classification [2], these tumors are categorized with a strong emphasis on molecular profiling and are divided into MB and other central nervous system (CNS) embryonal tumors [3]. Although pineoblastoma (PB) is no longer categorized within the CNS embryonal tumor group in the WHO 2021 classification, these patients are treated under the same regimens as other embryonal tumors.

Initial strategies involving conventional chemotherapy enabled the deferral of radiotherapy (RT) in a subset of patients; however, survival rates remained suboptimal, with most patients ultimately requiring salvage RT [4, 5]. Subsequently, high-dose chemotherapy (HDCT) with autologous stem cell rescue (ASCR) has been employed to reduce the severe long-term toxicities associated with RT and improve survival outcomes [6, 7]. Although some progress has been achieved, outcomes vary considerably across different MB molecular subgroups and non-MB embryonal tumors [8, 9].

This study aims to describe the clinical and molecular outcomes of a cohort of children prospectively treated with a transplant-based strategy at a single center in an upper-middle-income country, and to evaluate the feasibility of implementing such an approach in a resource-limited setting.

## Patients and methods

This prospective study was conducted between 2016 and 2019 at the Pediatric Oncology Institute–GRAACC, Federal University of São Paulo, and included children under 5 years of age diagnosed with CNS embryonal tumors, who had not received prior radiotherapy or chemotherapy. Data were analyzed in February 2025.

### Treatment protocol

Patients with less than 5 years of age, newly diagnosed CNS embryonal tumors, no prior RT or chemotherapy were included. Eligibility criteria and evaluations included in the protocol are summarized in Supplemental Material S1. Staging and tumor response were assessed according to the Chang system [11] and RECIST criteria [12], respectively The treatment plan consisted of maximal safe surgical resection followed by three to five cycles of induction chemotherapy based on a modified Head Start III protocol [10]—all cycles included high-dose methotrexate followed by cisplatin, cyclophosphamide, vincristine and etoposide, without temozolomide—followed by consolidation with one cycle of HDCT and ASCR consisting of carboplatin and thiotepa. Second-look surgery was considered for patients with residual disease on imaging after completion of induction chemotherapy. Although the original Head Start III protocol prescribed five fixed induction cycles, our modified protocol allowed three to five cycles based on response assessment, with consolidation proceeding after achieving complete response and successful stem cell collection following a minimum of three cycles and second-look surgery. This adaptation was designed to avoid unnecessary additional toxicity in early responders while maintaining the core transplant-based consolidation strategy of the Head Start III framework.

### Molecular assessment

All molecular assessments were done retrospectively due to the availability of the methods at the time of the study. This retrospective labeling does not imply a prospective molecular-based treatment decision at enrollment. The WHO 2021 classification was chosen for reporting outcomes to align with the most recent classification system.

#### Next-generation sequencing (NGS) using the Oncomine Childhood Cancer Research Assay® (OCCRA) panel

Fresh-frozen tumor samples were analyzed using targeted sequencing with the Oncomine™ Childhood Cancer Research Assay (OCCRA; Chef-ready, Thermo Fisher Scientific), which covers 255 tumor-associated genes, as previously reported [13].

#### Medulloblastoma molecular classification by nanostring

Patients with MB were classified according to the WHO 2021 classification into the following molecular subgroups: WNT-activated medulloblastoma, SHH-activated medulloblastoma with TP53 mutation, SHH-activated medulloblastoma with wild-type TP53, and non-WNT/non-SHH medulloblastoma, the latter further subdivided into Groups 3 and 4. Gene expression analysis was performed using the nCounter® FLEX Analysis System and a custom nCounter® Elements panel (NanoString Technologies, Seattle, WA, USA), as previously described [14–16].

For patients with MB in whom molecular subgrouping was unavailable due to inconclusive laboratory analyses, molecular classification was inferred based on clinicopathologic and imaging features, as previously described [17–19]. See Supplemental Material S2 for a complete description of molecular assessment.

#### Medulloblastoma molecular classification by clinicopathologic and imaging features

For patients with MB in whom molecular subgrouping was unavailable due to inconclusive laboratory analyses, molecular classification was inferred based on clinicopathologic and imaging features, as previously described (15–17). The SHH subgroup was defined by the presence of desmoplastic/nodular (DN) or extensive nodularity (EN) histology, positive GAB1 and/or YAP1 immunohistochemical staining, and a cerebellar mass located in the hemispheric or lateral region with contrast enhancement. Non-WNT/non-SHH tumors were defined by histology other than DN/EN, negative GAB1 and YAP1 immunohistochemical staining, and a centrally located cerebellar mass. Within this group, tumors with contrast enhancement were classified as Group 3, whereas tumors without contrast enhancement were classified as Group 4.

### Statistical analysis

EFS was defined as the time from diagnosis to disease progression, relapse, occurrence of a second neoplasm, or death from any cause, whichever occurred first. OS was defined as the time from diagnosis to death from any cause or last follow-up. Survival curves were estimated using the Kaplan–Meier method. Differences between survival curves were assessed using the log-rank test. All statistical analyses were performed using SPSS (v 30.0). A two-tailed p-value of less than 0.05 was considered statistically significant.

## Results

### Patients’ characteristics

Thirty-six patients were included: 22 with MB, six with AT/RT, one with PB, and seven with CNS embryonal tumors NOS. Of the latter, two were further classified based on methylation profiling as CNS neuroblastoma, FOXR2-activated, and CNS tumor with BCOR internal tandem duplication.

Among patients with MB, the mean age at diagnosis was 2.5 years (range, 1.5–4.8 years), and 13 were male. The mean interval from symptom onset to diagnosis was 3.9 months (range, 0.2–12 months). Gross total resection was achieved in 18 patients, and 15 had non-metastatic disease (M0).

Among patients with non-medulloblastoma tumors, the mean age at diagnosis was 2.7 years (range, 0.7–2.9 years), and 11 were female. The mean interval from symptom onset to diagnosis was 1.5 months (range, 0.5–5 months). Five were in the infratentorial compartment, and nine were in the supratentorial. Gross total resection was achieved in nine patients, and 12 had non-metastatic disease (M0).

### Molecular analyses

Molecular subgroup classification was successfully performed in 14 MB cases (63.6%) using the nCounter platform. Five cases yielded inconclusive results due to insufficient tumor material (Table 1). An additional eight cases were classified based on clinicopathologic and imaging features, as described in the “Methods” section: four were classified as SHH and three as Group 4. In one patient, only computed tomography imaging was available at diagnosis due to emergency neurosurgery, precluding distinction between Groups 3 and 4.

**Table 1:** Patient’s characteristics—medulloblastoma

ID	Sex	Age	Histology	Staging	Nanostring	Histopathology/MRI characteristics*	NGS (OCCRA)
1	F	4.5	Classic	R0M1	Inconclusive	Non WNT/SHH	*MET* (CNA); *BRAF* (CNA)
2	M	1.9	Classic	R0M0	SHH	-	Absence of variants
3	M	3	Classic	R0M0	Gr 3	-	Absence of variants
4	F	1.5	Classic	R0M0	SHH	-	*CDH7* (INDEL)
5	M	2.4	EN	R + M3	SHH	-	Absence of variants
6	F	4.2	DN	R + M1	SHH	-	Absence of variants
7	M	3.1	Classic	R0M1	Gr 4	-	Absence of variants
8	F	2.7	DN	R0M1	-	SHH	-
9	M	4.5	DN	R + M0	SHH	-	Absence of variants
10	F	4	DN	R0M0	Inconclusive	SHH	Absence of variants
11	F	3.3	Classic	R0M1	Gr 3	-	Absence of variants
12	F	2.5	DN	R0M0	SHH	-	*PTCH1* (INDEL); *CREBBP* (SNV); *SETBP1* (SNV); *SMARCB1* (SNV)
13	F	1.5	DN	R0M0	Inconclusive	SHH	Absence of variants
14	M	2.5	Classic	R0M0	SHH	-	*PIK3CA* (SNV); *PTCH1*(INDEL)
15	M	1.4	Classic	R + M0	Gr 3	-	Absence of variants
16	M	2.6	DN	R0M0	SHH	-	Absence of variants
17	M	2.3	DN	R0M0	-	SHH	-
18	M	1.9	Classic	R0M0	-	Gr 4	-
19	M	4.8	DN	R0M3	SHH	-	Absence of variants; *TP53* (FISH)^#^
20	M	2.6	Classic	R0M0	Inconclusive	Gr 4	-
21	M	1.8	Classic	R0M0	Inconclusive	Gr 4	-
22	F	2	Classic	R0M0	Gr 3	-	-

*CNA* copy number alterations, *DN* desmoplastic/nodular, *EN* extensive nodularity, *F* female, *Gr* group, *INDEL* insertions and deletions, *M* male, M0-3 metastatic staging according to Chang et al., *R0* gross total resection, *R* + subtotal resection > 1.5 m^2^, *SHH* sonic hedgehog, *SNV* single-nucleotide variants

^*^Cases classified with histopathology/MRI characteristics according to methods

^#^TP53–c.818G > A, p.(Arg273His) Fluorescence in situ hybridization (FISH)

All DNMB and MBEN tumors and 3 of 12 classic MB cases (25%) were classified as belonging to the SHH subgroup, which accounted for 13 of 22 cases (59.1%). Non-WNT/non-SHH tumors comprised nine of the 22 cases (40.9%), including four classified as Group 3 and four as Group 4. Among patients with metastatic disease at diagnosis, 4 of 7 cases (57.1%) belonged to the SHH subgroup.

NGS successfully identified genetic variants in 4 of the 16 patients analyzed. In two cases, the findings supported the molecular classification, including the identification of a PTCH1 variant in a tumor classified as SHH medulloblastoma (Table 1).

Among non-medulloblastoma tumors, methylation profiling was available for two patients, as previously described (CNS neuroblastoma, FOXR2-activated, and CNS tumor with BCOR internal tandem duplication). Of the nine patients analyzed by NGS, genetic variants were identified in only one patient diagnosed with AT/RT (Table 2). Patient characteristics and molecular findings are summarized in Tables 1 and 2.

**Table 2:** Patient’s characteristics—non-medulloblastoma

ID	Sex	Age	Diagnosis	Staging	NGS (OCCRA)
23	M	1	AT/RT	R0M1	Absence of variants
24	F	0.8	AT/RT	R0M1	Absence of variants
25	F	0.8	AT/RT	R + M0	Absence of variants
26	F	1	AT/RT	R + M0	Absence of variants
27	F	11	AT/RT	R + M0	Absence of variants
28	F	8	AT/RT	R0M0	*CIC* (SNV); *SMARCB1*(SNV)
29	M	2.8	BCOR*	R0M0	-
30	F	2.9	Embryonal NOS	R + M0	-
31	F	2.2	Embryonal NOS	R0M0	-
32	M	1.1	Embryonal NOS	R0M0	-
33	F	1.7	Embryonal NOS	R0M0	Absence of variants
34	F	2	Embryonal NOS	R0M0	Absence of variants
35	F	2	NBL FOXR2*	R + M0	-
36	F	0.7	Pineoblastoma	R0M0	Absence of variants

*AT*/*RT* atypical teratoid/rhabdoid tumor, *F* female, *M* male, M0-3 metastatic staging according to Chang et al., *NOS* not otherwise specified, *R0* gross total resection, *R* + subtotal resection > 1.5 m^2^, *SNV* single-nucleotide variants,

^*^Methylation profile

### Treatment outcome

#### Medulloblastoma cohort

The median time from surgery to initiation of induction chemotherapy was 42.5 days (range, 15–180 days). The prolonged delays in some patients were primarily attributable to system-related factors, including logistical barriers related to patient transfer from referring institutions and patient-related factors, such as infectious complications in the postoperative period. As shown in Table 3, time to start induction did not significantly affect survival outcomes in this cohort. All but two patients with MB completed three to five cycles of induction chemotherapy. Two patients with MB died during induction therapy: one due to progressive disease after three cycles and one due to septic shock after two cycles. At the completion of the induction and consolidation phases, all eligible patients (*n* = 20) achieved complete remission.

**Table 3:** Prognostic factor in medulloblastoma

	5-year EFS (%)	*p*-value	5-year OS (%)	*p*-value
Sex				
F	55.6%	0.93	77.8%	0.95
M	52.7%		76.9%	
Time to start induction				
< 30 days	42.9%	0.42	71.4%	0.65
> 30 days	60.0%		79.4%	
Extent of resection				
GTR	50.0%	0.42	72.2%	0.27
STR	75.0%		100%	
Metastases				
M0	66.7%	0.13	80.0%	0.73
M+	28.6%		68.6%	
Histology				
Desmoplastic/MBEN	70.0%	0.18	90.0%	0.20
Classic	41.7%		66.7%	
Molecular subgroup				
SHH	76.9%	0.008	92.3%	0.043
Non-WNT/non-SHH	22.2%		55.6%	
Induction cycles				
3	33.3%	0.32	83.3%	0.47
4	83.3%		100%	
5	62.5%		75.0%	

*EFS* event free survival, *F* female, *GTR* gross total resection, *M* male, *M0* no metastases, *M* + metastases M1–M3 according to Chang et al., *MBEN* medulloblastoma with extensive nodularity, *OS* overall survival, *SHH* sonic hedgehog, *STR* subtotal resection > 1.5 m^2^

**Fig. 1:**
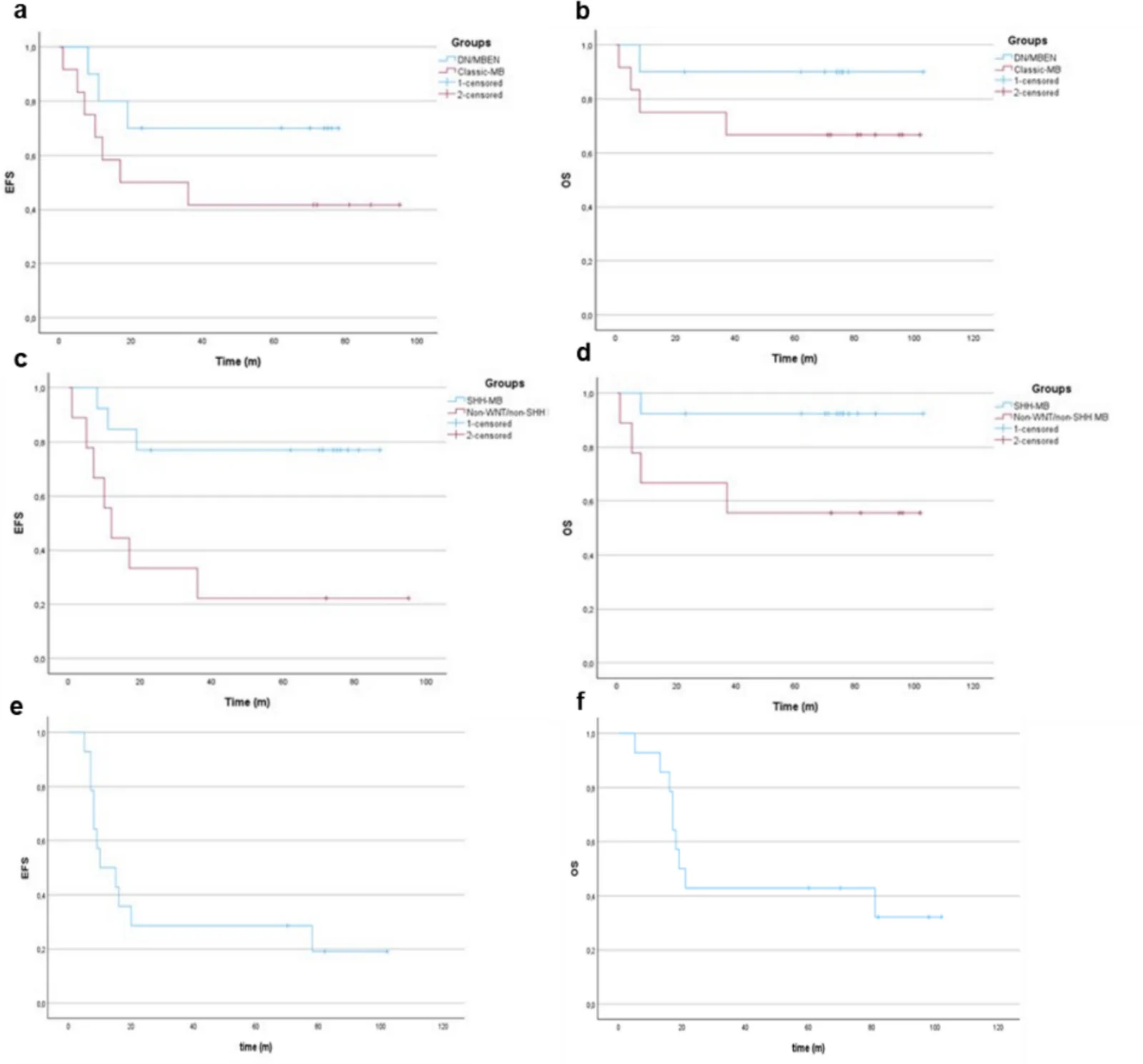
Kaplan–Meier curves of event-free survival (EFS) and overall survival (OS) for patients with medulloblastoma (**a**–**d**) and non-MB (**e**–**f**): a event-free survival according to histologic subtype; blue line, desmoplastic/nodular and extensive nodularity MB; red line, classic-MB; **b** Overall survival according to histologic subtype; blue line, desmoplastic/nodular and extensive nodularity MB; red line, classic-MB; **c** Event-free survival according to molecular subgroups; blue line, SHH; red line, non-WNT/non-SHH;** d** Overall survival according to molecular subgroups; blue line, SHH; red line, non- WNT/non-SHH; e event-free survival for non-medulloblastoma tumors; and f overall survival for non-medulloblastoma tumors

According to histologic subtype, the 2- and 5-year EFS rates for DN/MBEN were 70% (95% CI, 41.6–98.4%), and the OS rate was 90% (95% CI, 71.4–100%). For classic MB, the 2- and 5-year EFS rates were 50% and 41.7% (95% CI, 13.9–69.5%), respectively, while OS rates were 75% and 66.7% (95% CI, 40.0–93.4%), respectively (Fig. 1a and b). Differences according to histologic subtype did not reach statistical significance (EFS, *p* = 0.185; OS, *p* = 0.205).

According to the molecular subgroup, the 2- and 5-year EFS rates for the SHH subgroup were 76.9% (95% CI, 54.0–99.8%), and the OS rate was 92.3% (95% CI, 77.8–100%). In contrast, for the non-WNT/non-SHH subgroup, the 2- and 5-year EFS rates were 33.3% and 22.2% (95% CI, 0–49.4%), respectively, while the corresponding OS rates were 66.7% and 55.6% (95% CI, 23.0–88.1%), respectively (Fig. 1c and d). Differences according to molecular subgroup were statistically significant for both EFS (log-rank *p* = 0.008) and OS (log-rank *p* = 0.043).

The patient who died during induction therapy due to progressive disease (ID 21) had R0M0 staging, classic histology, and was molecularly classified as non-WNT/non-SHH (Group 4).

Among patients who experienced relapse, two with SHH MB (IDs 6 and 19) relapsed. Both demonstrated p53 overexpression (> 50%) on immunohistochemistry and had metastatic disease at diagnosis (R + M1 and R0M3, respectively). One patient (ID 19) had a TP53 alteration confirmed by fluorescence in situ hybridization (FISH). The first patient developed craniospinal relapse, and the second developed local relapse at 11 and 19 months after diagnosis, respectively. Both patients were successfully treated with craniospinal irradiation (CSI) with 36 Gy after the age of 5 years and remain alive, with overall survival durations of 76 and 103 months, respectively.

One additional patient with SHH MB (ID 16) died 4 months after consolidation due to meningitis, without evidence of disease recurrence.

Five patients with non-WNT/non-SHH MB experienced relapse: three had local relapse (IDs 1, 3, and 20) at 10, 17, and 36 months after diagnosis, respectively, and two had distant relapse (IDs 7 and 11) at 7 and 12 months after diagnosis, respectively. All were treated with 36 Gy CSI. Patients with local relapse remain alive at 38, 82, and 95 months of follow-up, respectively, whereas the two patients with distant relapse died at 8 and 37 months after diagnosis.

### Prognostic factors

Prognostic factors for the MB cohort are summarized in Table 3. EFS and OS differed significantly according to the molecular subgroup. Patients with SHH MB had significantly better outcomes compared with those with non-WNT/non-SHH tumors (5-year EFS for the SHH subgroup was 76.9% versus 22.2% for the EFS non-WNT/non-SHH, *p* = 0.008 and 5-year OS 92.3% versus 55.6%, respectively, *p* = 0.043). Histologic subtype and metastatic status were not significantly associated with survival outcomes, likely due to the small sample size.

#### Non-MB cohort

The median time from surgery to initiation of induction chemotherapy was 22 days (range, 4–91 days). All patients in this group completed three to five cycles of induction chemotherapy. Treatment response at the end of induction and consolidation was evaluated in 14 patients and is summarized in Table 4.

**Table 4:** Number of Induction cycles and disease status after induction and consolidation therapy

	***N***	**%**
**Induction cycles**		
3	8/14	57.1%
4	1/14	7.1%
5	4/14	28.6%
**Status after induction (response)**		
CR	10/14	71.4%
PR	4/14	28.6%
**Status after consolidation (response)**		
CR	10/14	71.4%
PR	4/14	28.6%

*CR* complete response, *PR* partial response

Among the four patients with PR after induction chemotherapy, three had AT/RT (IDs 25, 26, and 27), and one had an embryonal tumor, NOS, located in the brainstem (ID 30). Second-look surgery was not performed in these cases due to unresectable brainstem lesions. All patients with PR received radiotherapy after consolidation, including two who received focal radiotherapy with 50–54 Gy and two who received CSI 36 Gy. Additionally, the patient with a CNS tumor with BCOR internal tandem duplication (ID 29), who achieved CR, received focal radiotherapy with 54 Gy.

All patients with AT/RT experienced relapses (three local, two spinal, and one combined) and received second-line chemotherapy followed by CSI with 36 Gy. However, only one patient (ID 24) remains alive, with an overall survival of 98 months after diagnosis.

Among patients with non-AT/RT embryonal tumors, one patient with an embryonal tumor, NOS (ID 30), had a PR after induction, as previously described, received CSI with 36 Gy, and died 17 months after diagnosis. The patient with a CNS tumor with BCOR internal tandem duplication (ID 29) relapsed 7 months after diagnosis despite focal radiotherapy and died 17 months after diagnosis. The patient with PB (ID 36) remains alive without relapse at 102 months of follow-up. The patient with CNS neuroblastoma, FOXR2-activated (ID 35), relapsed 20 months after diagnosis, received 36 Gy CSI, and remained alive with stable disease and an overall survival of 60 months.

Among patients with embryonal tumor, NOS, one of four patients (ID 34) relapsed 8 months after diagnosis and died at 21 months. One patient (ID 31) was lost to follow-up, while the remaining two patients (IDs 32 and 33) remain alive without evidence of disease, with overall survival durations of 70 and 82 months, respectively.

For all non-medulloblastoma tumors, the 2- and 5-year EFS rates were 28.6% and 19.0% (95% CI, 0%–41.0%), respectively, and the OS rates were 42.9% and 32.1% (95% CI, 5.4%–58.8%), respectively (Fig. 1e and f). When analyzing AT/RT separately, the 2- and 5-year EFS rates were 16.7% and 0%, respectively, while the corresponding OS rates were 33.3% and 16.7% (95% CI, 0%–46.5%), respectively. Excluding patients with AT/RT, the 2- and 5-year EFS rates were both 37.5% (95% CI, 4.0%–71.0%), while the corresponding OS rates were both 50.0% (95% CI, 15.3%–84.7%), respectively.

### Toxicity

The most common toxicities in the MB group were gastrointestinal complications and fever. All patients developed grade 3–4 mucositis and/or typhlitis and febrile neutropenia during HDCT and ASCR. Invasive fungal infections occurred in 4 of 22 patients (18.2%) during the induction phase, including bloodstream infections caused by *Candida tropicalis*, *Candida parapsilosis*, *and Candida albicans*, as well as one case of probable invasive pulmonary aspergillosis. As previously described, one patient died due to septic shock during induction chemotherapy. During consolidation, two patients developed veno-occlusive disease (VOD), and five developed bloodstream infections.

Among patients with non-medulloblastoma tumors, one patient (7.1%) developed an invasive fungal infection during induction (bloodstream infection caused by *Candida parapsilosis*). Febrile neutropenia occurred in 12 of 14 patients, including three cases of typhlitis, and 7 of 14 patients developed bloodstream infections during ASCR. No thiotepa-related neurotoxicity or treatment-related deaths occurred during the consolidation phase.

## Discussion

Children younger than 5 years of age with CNS embryonal tumors can be treated with a multimodal strategy based on surgery, conventional chemotherapy, and consolidation with HDCT using thiotepa and ASCR, resulting in variable outcomes, as demonstrated in the present study.

EFS differed significantly according to histologic subtype and molecular subgroup within the MB cohort. Patients with MBEN/DN tumors achieved a 5-year EFS of 70%, comparable to the outcomes reported in the Head Start III study [10], which included both localized and metastatic DN/MBEN cases and reported a 5-year EFS and OS of 89% ± 6%, with an irradiation-free EFS of 78% ± 8% specifically for DN tumors. In contrast, the HIT-2000 study [20] reported a higher 5-year EFS of 93% in patients treated with intraventricular and high-dose intravenous methotrexate combined with conventional chemotherapy; however, that cohort included only non-metastatic patients. Importantly, in our cohort metastatic status was not significantly associated with survival outcomes, likely due to the limited sample size.

Excluding the patient who died due to infection, both patients who relapsed were older than 4 years at diagnosis and showed evidence suggestive of TP53 alteration, based on p53 overexpression on immunohistochemistry, with one case confirmed by molecular analysis. Germline TP53 testing was not performed. These findings may partially explain the poorer survival observed, as TP53 alterations are associated with a higher risk of treatment failure in SHH MB [21]. Both patients were successfully salvaged with craniospinal irradiation (CSI), although the optimal management strategy for this subgroup remains under investigation [22, 23, 24].

In our cohort, patients with classic MB had a 5-year EFS of 41.7%, which was higher than that reported in the Head Start III study [10], where the 5-year EFS was 26% for children with classic MB (*n* = 26) and 38% for those with large cell/anaplastic (LCA) histology (*n* = 13). Similarly, the CCG-99703 study [25] reported a 5-year EFS of approximately 50% (*n* = 18) using a regimen consisting of three cycles of induction chemotherapy followed by three cycles of high-dose consolidation chemotherapy with thiotepa and carboplatin. The German HIT group reported a 5-year EFS of 37% among children with classic MB and LCA histology (*n* = 45) [20].

Due to the small sample size in our cohort, formal statistical analysis of prognostic factors was limited. Overall, our findings are consistent with previously published studies showing modest survival outcomes for this subgroup. As highlighted by Bouffet et al*.* [8], despite multiple therapeutic strategies, including intensified chemotherapy and consolidation with high-dose regimens, no clearly superior treatment approach has yet been established for children with non-SHH/WNT MB.

Regarding molecular classification, our findings are consistent with the known epidemiology of medulloblastoma in infants. Approximately two-thirds of cases in this age group are associated with SHH pathway activation, while the remaining cases are predominantly Group 3 and, less commonly, Group 4 tumors, with WNT-activated medulloblastoma being exceedingly rare [26, 27].

As expected, all DN/MBEN cases in our cohort belonged to the SHH subgroup. Notably, 3 of 12 patients (25%) with classic histology were also classified within the SHH subgroup, and all of these patients remain alive without disease recurrence. These findings further support the favorable prognosis associated with SHH medulloblastoma in young children, regardless of histologic subtype. However, the existence or clinical impact is still questionable [9, 28].

Consistent with recently reported molecular subgroup-specific outcomes, patients in the SHH subgroup demonstrated excellent survival and represent an important prognostic group when treated with HDCT/ASCR or intrathecal/intraventricular methotrexate-based strategies. These approaches may mitigate the prognostic differences previously observed between SHH subtypes, such as SHH-1 and SHH-2, in the ACNS1221 [29] and SJYC07 [30] trials, particularly in radiation-sparing protocols that did not include intraventricular chemotherapy. A limitation of our study is the incomplete availability of molecular subgrouping data, which precluded more detailed subgroup-specific outcome analyses.

The management of young children with non-medulloblastoma embryonal tumors remains a major clinical challenge. AT/RT, although accounting for approximately 3% of all pediatric CNS tumors, is the most common malignant CNS tumor in children younger than 1 year of age and accounts for up to 20% of cases in those younger than 3 years [31]. In our cohort, AT/RT represented 85.7% of tumors diagnosed in children younger than 1 year of age.

Current molecular classification identifies three epigenetic subgroups of AT/RT—TYR, SHH, and MYC [31–33]. Although definitive prognostic correlations remain limited, some studies suggest that the TYR subgroup may be associated with more favorable outcomes, particularly in infants [33, 34]. In our study, despite the lack of molecular subgrouping, patients with AT/RT had dismal outcomes, regardless of the use of radiotherapy either as part of salvage treatment or at relapse. These findings are consistent with previously reported survival outcomes, which remain poor despite intensive multimodal treatment strategies [35–37].

Other embryonal tumors, including PB, ETMR, CNS neuroblastoma, FOXR2-activated, and CNS tumors with BCOR internal tandem duplication, are generally associated with poor prognosis. A recent meta-analysis by Mynarek et al*.* [38] reported an overall survival rate of 12% in children younger than 4 years with PB. Similarly, reported 5-year survival rates for ETMR range from 0 to 30% [39, 40]. Historical series of supratentorial embryonal tumors have shown 5-year EFS rates of approximately 39% [41].

Clinical data on molecularly defined rare CNS embryonal tumors remain limited, highlighting the importance of molecular diagnostic classification to improve prognostic stratification and guide therapeutic decision-making [42, 43]. In our cohort, due to the small number of patients and incomplete availability of methylation profiling, survival analyses were performed by grouping non-medulloblastoma tumors, excluding AT/RT. In this subgroup, the 5-year EFS and OS rates were 37.5% and 50%, respectively.

NGS has enabled the identification of novel molecular markers, signaling pathways, and predictive biomarkers, facilitating the detection of actionable alterations and supporting the development of personalized therapeutic approaches in pediatric medulloblastoma and other embryonal CNS tumors [44–48]. Furthermore, certain somatic alterations may indicate an underlying germline predisposition syndrome [47–49]. Examples include *PTCH1* and *SUFU* mutations associated with Gorlin syndrome and *TP53* alterations associated with Li–Fraumeni syndrome in patients with SHH-activated medulloblastoma, as observed in our cohort, as well as *DICER1* mutations in pineoblastoma and *SMARCB1* or *SMARCA4* alterations in AT/RT [50, 51].

Although germline testing was not systematically performed in our study, most patients were evaluated and followed by a clinical geneticist for surveillance and genetic counseling, highlighting the importance of integrating molecular findings into comprehensive patient management.

Treatment-related toxicities, primarily grade 3–4 hematologic and gastrointestinal adverse events, as well as episodes of febrile neutropenia, were consistent with those reported in other ASCR-based studies [41, 52]. In HSIII [10], grades 3–4 toxicities during induction and consolidation were primarily related to myelosuppression, infection, mucositis, and electrolyte abnormalities, with two toxic deaths reported—one from severe mucositis and gastrointestinal hemorrhage during induction, and one from VOD and multisystem organ failure during consolidation. In our cohort, all patients developed grades 3–4 mucositis and/or typhlitis and febrile neutropenia; two patients developed VOD during consolidation; five patients developed bloodstream infections during ASCR; and one toxic death occurred during induction due to septic shock. The most notable divergence, as previously reported by our group [53], was the higher invasive fungal disease rate observed in our cohort, whereas 5 of 36 patients (13.9%) developed documented invasive fungal infections during HDCT, most commonly *Candida* spp. bloodstream infections. As discussed in the previous paper [53], routine systemic antifungal prophylaxis based solely on diagnosis is not recommended. However, it is important to assess individual patient- and treatment-related risk factors. In cases involving infants undergoing high-dose chemotherapy with profound and prolonged neutropenia, agents such as fluconazole represent a suitable choice, particularly in LMICs, due to their favorable cost-effectiveness, low potential for drug interactions, and manageable side-effect profile.

Although preservation of neurocognitive function is a major rationale for radiation-sparing treatment strategies in young children with brain tumors [28], a limitation of our study is the lack of systematic assessment of long-term neurocognitive outcomes. This reflects constraints related to limited multidisciplinary resources and financial support, which are common challenges in middle-income settings. Future studies in similar settings should consider incorporating feasible neurodevelopmental screening tools adapted to resource-limited environments, such as the Ages and Stages Questionnaires (ASQ) or the Pediatric Evaluation of Disability Inventory (PEDI). Collaboration with local psychologists and incorporation of structured parental-report instruments could provide meaningful neurodevelopmental data even in the absence of formal neuropsychological testing infrastructure.

## Conclusion

HDCT/ASCR is feasible in well-structured centers, even in middle-income countries such as Brazil. Patients with SHH medulloblastoma had favorable outcomes, supporting this radiation-sparing strategy in young children. In contrast, non-SHH medulloblastoma and other CNS embryonal tumors remain associated with poor prognosis, highlighting the need for improved molecularly guided therapies.

## Supplementary Information

Below is the link to the electronic supplementary material.

ESM 1 (DOCX 84.5 KB)

ESM 2 (DOCX 15.0 KB)

## Data Availability

The datasets generated during and/or analyzed during the current study are available from the corresponding author upon reasonable request.
