# Supplementary material for: High-dose chemotherapy with autologous stem cell rescue in children under 5 years of age with central nervous system embryonal tumors: results from a prospective cohort in an upper-middle-income country
Source: Childs Nerv Syst. 2026 Jun 30;42(1):274. doi: 10.1007/s00381-026-07367-w (PMC13319136; doi:10.1007/s00381-026-07367-w)
Supplement: Supplementary file 1 — (DOCX 84.5 KB) [file 381_2026_7367_MOESM1_ESM.docx]

**Treatment Protocol**

***Eligibility criteria***

Patients younger than five years of age with newly diagnosed CNS embryonal tumors who had not received prior radiotherapy or chemotherapy, except for corticosteroids, and had pathology-confirmed medulloblastoma, AT/RT, ETMR, PB, or embryonal tumor not otherwise specified (NOS) were included.

***Staging***

Initial staging consisted of craniospinal MRI with and without contrast administration and lumbar cerebrospinal fluid (CSF) analysis, unless clinically contraindicated, performed at diagnosis or at least 14 days after surgery. Metastatic disease was assessed using the Chang staging system (10). The extent of resection was classified as biopsy only, gross total resection (residual tumor <1.5 cm²), or subtotal resection (residual tumor ≥1.5 cm²).

***Pathology and Image characteristics and review***

All histopathologic diagnoses and pre- and postoperative magnetic resonance imaging (MRI) studies were reviewed by an institutional neuropathologist and a neuroradiologist.

***Evaluation and Follow-up***

Craniospinal MRI with and without gadolinium contrast and lumbar CSF analysis were performed after every two cycles of chemotherapy, after ASCR, and subsequently every 4 months during the first 2 years, every 6 months until 5 years after completion of therapy, and annually thereafter. Tumor response was assessed according to RECIST criteria (19).

***Induction phase***


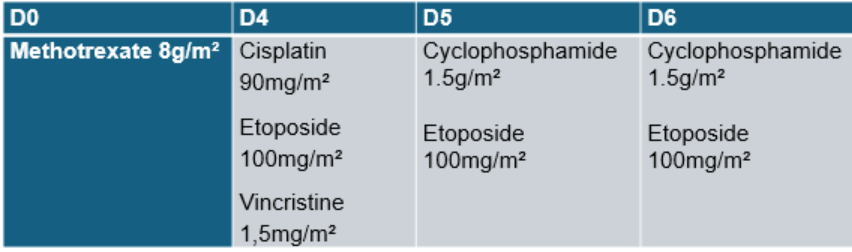


*D4 starts only after MTX levels < 0.3μmol/L

The induction chemotherapy regimen consisted of three to five cycles administered every 3 weeks.

- Folinic acid rescue (15 mg/m²), urine alkalinization, and serial monitoring of serum methotrexate levels were performed during the first phase. The second phase was initiated only after methotrexate levels decreased to <0.3 μmol/L.
- Granulocyte colony-stimulating factor (G-CSF) was administered at a dose of 5 mcg/kg/day subcutaneously until the post-nadir white blood cell count reached 10,000/mm³. Before each cycle, the following hematologic and biochemical criteria were required: absolute neutrophil count ≥500/mm³, platelet count ≥100,000/mm³, serum creatinine ≤1.5 mg/dL, transaminases ≤5 times the institutional upper limit of normal, and total bilirubin ≤2.0 mg/dL. Treatment was delayed if these criteria were not met; however, episodes of febrile neutropenia or delayed hematologic recovery did not require dose reductions in subsequent cycles.
- Peripheral blood stem cells were collected after the first, second, or third cycle of induction chemotherapy.
- Second-look surgery was considered in patients with residual disease on imaging after completion of induction chemotherapy. Patients without evidence of tumor progression during induction chemotherapy proceeded to ASCR.

***Consolidation Phase***

All eligible patients received one cycle of high-dose chemotherapy followed by ASCR, consisting of carboplatin (17 mg/kg/day on days −4 and −3) and thiotepa (10 mg/kg/day on days −4 and −3).

- G-CSF was administered starting on day +1 and continued until the absolute neutrophil count exceeded 2,000/mm³ for two consecutive days.
- Ursodiol was initiated at admission and continued through day +90. All blood products were irradiated and leukocyte-depleted.
- Patients were managed in rooms equipped with high-efficiency particulate air filtration.

***Toxicity***

Adverse events were graded according to the Common Terminology Criteria for Adverse Events, version 4.0.
