# Supplementary material for: High-dose chemotherapy with autologous stem cell rescue in children under 5 years of age with central nervous system embryonal tumors: results from a prospective cohort in an upper-middle-income country
Source: Childs Nerv Syst. 2026 Jun 30;42(1):274. doi: 10.1007/s00381-026-07367-w (PMC13319136; doi:10.1007/s00381-026-07367-w)
Supplement: Supplementary file 2 — (DOCX 15.0 KB) [file 381_2026_7367_MOESM2_ESM.docx]

**Molecular profiling techniques**

***Next-generation sequencing using the Oncomine Childhood Cancer Research Assay® (OCCRA) panel***

Total DNA and RNA were extracted from FFPE (formalin-fixed, paraffin-embedded) samples using the MagMAX™ FFPE DNA/RNA Ultra Kit (Applied Biosystems), and from frozen tumor samples using the FastPrep®-24 Tissue Homogenizer (MP Biomedicals®) and the AllPrep DNA/RNA Mini Kit (QIAGEN®), both according to the manufacturers’ instructions. Isolated DNA and RNA were quantified using a NanoDrop 2000® spectrophotometer, Qubit 3® fluorometer, and real-time PCR.

After quantification, complementary DNA (cDNA) synthesis was performed using 20 ng of total RNA according to the manufacturer’s protocol for SuperScript™ IV VILO® (Thermo Fisher Scientific®). Sequencing libraries were prepared from 20 ng of DNA and 20 ng of cDNA from each tumor sample, following the manufacturer’s protocol for the Oncomine Childhood Cancer Research Assay (OCCRA®; Thermo Fisher Scientific®), as previously reported (11). The OCCRA panel comprises 203 unique genes, including 130 DNA driver genes, 28 copy number variation targets, a fusion panel covering 90 driver genes with multiple partners, and 9 gene expression controls. These targets encompass the most relevant genomic alterations in pediatric and young adult cancers.

Amplicon libraries with sample-specific barcodes were generated from DNA and cDNA, and automated clonal amplification was performed using the Ion Chef™ System (Thermo Fisher Scientific®). Libraries were then loaded onto the Ion 540™ Chip and sequenced on the Ion S5™ System (Thermo Fisher Scientific®). Sequencing was performed using 200-bp read lengths, and sequencing data were processed using the manufacturer’s standard analysis pipeline.

Sequencing quality was assessed using Torrent Suite™ software version 5.2.1 (Thermo Fisher Scientific®). Only amplicon reads with a forward-to-reverse primer ratio between ≥0.6 and ≤1.4 were included. Reads were aligned to the hg19/GRCh37 human reference genome using Torrent Suite™ software, and only reads with a minimum coverage depth of 2000× were considered for analysis. The resulting BAM files were visualized and analyzed using Integrative Genomics Viewer (IGV; Broad Institute, Cambridge, MA, USA). Variant Call Format (VCF) files were generated, and identified variants were annotated and compared with the COSMIC, dbSNP, and ExAC databases.

Variants were classified according to variant type (single nucleotide variants [SNVs] or insertions/deletions [InDels]), functional effect (missense, nonsense, synonymous, frameshift, and non-frameshift), and clinical significance (pathogenic, likely pathogenic, variant of uncertain significance, likely benign, and benign). Pathogenicity classification was supported by FATHMM prediction scores ranging from 0 to 1, with scores ≥0.7 considered pathogenic. For copy number variation (CNV) analysis, only samples with a median absolute pairwise difference (MAPD) ≤0.35 were included. Variant interpretation was further supported using the COSMIC, ClinVar, VarSome, UCSC Genome Browser, and Ensembl Genome Browser databases.

***Medulloblastoma molecular classification by Nanostring***

Using the nCounter® (NanoString) platform, patients with MB were classified according to the WHO 2021 classification into the following molecular subgroups: WNT-activated medulloblastoma, SHH-activated medulloblastoma with TP53 mutation, SHH-activated medulloblastoma with wild-type TP53, and non-WNT/non-SHH medulloblastoma, the latter further subdivided into Group 3 and Group 4.

Briefly, following total RNA isolation from FFPE samples, gene expression analysis was performed using the nCounter® FLEX Analysis System and a custom nCounter® Elements panel (NanoString Technologies, Seattle, WA, USA), as previously described (12–14). The panel included three reference genes (ACTB, GAPDH, and LDHA) and 22 target genes used to classify molecular subgroups: WNT pathway (WIF1, TNC, GAD1, DKK2, and EMX2), SHH pathway (PDLIM3, EYA1, HHIP, ATOH1, and SFRP1), Group 3 (IMPG2, GABRA5, EGFL11, NRL, MAB21L2, and NPR3), and Group 4 (KCNA1, EOMES, KHDRBS2, RBM24, UNC5D, and OAS1).

***Medulloblastoma molecular classification by* clinicopathologic and imaging features**

For patients with MB in whom molecular subgrouping was unavailable due to inconclusive laboratory analyses, molecular classification was inferred based on clinicopathologic and imaging features, as previously described (15–17).

The SHH subgroup was defined by the presence of desmoplastic/nodular (DN) or extensive nodularity (EN) histology, positive GAB1 and/or YAP1 immunohistochemical staining, and a cerebellar mass located in the hemispheric or lateral region with contrast enhancement. Non-WNT/non-SHH tumors were defined by histology other than DN/EN, negative GAB1 and YAP1 immunohistochemical staining, and a centrally located cerebellar mass. Within this group, tumors with contrast enhancement were classified as Group 3, whereas tumors without contrast enhancement were classified as Group 4.
